# Synthesis of Low-Silicon X-Type Zeolite from Lithium Slag and Its Fast Exchange Performance of Calcium and Magnesium Ions

**DOI:** 10.3390/ma17133181

**Published:** 2024-06-28

**Authors:** Yu Wang, Longbin Deng, Lin Zhang, Qun Cui, Haiyan Wang

**Affiliations:** College of Chemical Engineering, Nanjing Tech University, Nanjing 211816, China; 202161104067@njtech.edu.cn (Y.W.); denglongbin@njtech.edu.cn (L.D.); linzhang@njtech.edu.cn (L.Z.); haiyanwang@njtech.edu.cn (H.W.)

**Keywords:** lithium slag, low-silicon X-type zeolite (KNaLSX), fast washing detergent builder, calcium and magnesium ion exchange, exchange rate constant

## Abstract

Without the addition of silicon and aluminum sources, a pure-phase KNaLSX zeolite was successfully synthesized from the residue (lithium slag), which was produced from spodumene in the production process of lithium carbonate. The KNaLSX samples were characterized by an X-ray Diffractometer (XRD), Scanning Electron Microscope (SEM), X-ray Fluorescence Spectrometer (XRF), Thermogravimetric Differential Thermal Analysis (TG-DTA), Fourier Transform Infrared Spectrometer (FT-IR), and N_2_ adsorption measurement. The ion exchange capacity and the ion exchange rate of calcium and magnesium ions were measured as used for a detergent builder, and the results were compared with the standard zeolites (KNaLSX and 4A). The experimental results show that the pure-phase KNaLSX synthSynthesis and characterization of co-crystalline zeolite composite of LSX/esized from lithium slag has a SiO_2_/Al_2_O_3_ ratio of 2.01 with a grain size of 3~4 μm, which is close to the commercial KNaLSX sample of a SiO_2_/Al_2_O_3_ ratio of 2.0. The BET-specific surface area of KNaLSX is 715 m^2^/g, which is larger than the low-silicon X-type zeolite (LSX) synthesized from waste residue reported in the literature. The ion exchange rate constant of calcium and magnesium ions in KNaLSX is 5 times and 3 times that of 4A zeolite, respectively. KNaLSX also has a high ion exchange capacity for magnesium ion of 191 mgMgCO_3_/g, which is 2 times than that of 4A zeolite, and a high ion exchange capacity for calcium ion of 302 mgCaCO_3_/g, which meets the first-grade standard of zeolite for detergent builders in China. The work provides the basis for high-value resource utilization of lithium slag and the development of a detergent builder for rapid washing.

## 1. Introduction

Lithium slag is the solid waste discharged in the production of lithium carbonate by the spodumene sulfuric acid process (named lithium slag), and the production of 1 ton of lithium carbonate produces 8–10 tons of lithium slag [1]. In China, lithium carbonate production was 517,900 tons in 2023, and there were over 4 million tons of lithium slag. Currently, only a small amount of lithium slag is used as cement or concrete admixture [2,3,4,5], cement ceramic material [6,7], geopolymer material [8,9,10], etc. Most of the lithium slag has not been effectively used. Therefore, high-value exploitation and utilization of lithium slag is of great significance to the sustainable development of lithium extraction enterprises from spodumene.

The content of SiO_2_ and Al_2_O_3_ in lithium slag is greater than 97% with a small amount of calcium, iron, and other impurities after pretreatment, and the SiO_2_/Al_2_O_3_ ratio of 3.5~4.8 [11,12,13], which is suitable for the synthesis materials of NaA (4A), NaX (13X), and NaY [14,15,16,17]. Currently, 4A, 13X, and NaY zeolites are widely used in petrochemical, biomedicine, daily chemical industries, and other industries due to their excellent adsorption, catalysis, and ion exchange capabilities. Among them, NaY faujasite zeolite is an important adsorbent for the removal of heavy metals (such as cobalt ions with adsorption capacity of 1.82 mmol/g) [18], but a mesoporous adsorbent based on calcium silicate CaSiO_3_ also has similar exchange capacity of heavy metals and can be substituted as adsorbents [19]. Further, 8–15% 4A zeolite as a detergent builder is usually added to laundry powder for softening water. In 2022, about 1.4 million tons of 4A zeolite were used as a detergent builder in the global market of zeolites, accounting for 65% of the total consumption of zeolites [20]. Here, 13X zeolite is usually used as an adsorbent and catalyst. Literature reports [21,22] that 13X zeolite has a pore size larger than that of 4A zeolite, which is conducive to hydrated magnesium ion exchange, and can be used as a washing builder to improve the magnesium ion exchange performance. However, the market price of 13X zeolite is expensive compared with that of 4A zeolite, and even a small amount of addition or partial replacement of 4A zeolite significantly increases the cost of detergent additives.

In recent years, the preparation of NaX (13X) zeolite using waste residue has attracted much attention. Ibsaine et al. [23] synthesized NaX zeolite with a calcium ion exchange capacity of 150 mgCaCO_3_/g using lithium slag as the raw material. Liu et al. [24] synthesized NaX zeolite with fly ash as a raw material, and its calcium ion exchange capacity was 119.5 mgCaCO_3_/g, and its magnesium ion exchange capacity was 119 mgMgCO_3_/g. Qi et al. [25] used gold mine tailing as the raw material to synthesize NaX zeolite, which had a calcium ion exchange capacity of 62.78 mgCaCO_3_/g and a magnesium ion exchange capacity of 12.56 mgMgCO_3_/g. Chandrasekhar et al. [26] synthesized NaX zeolite with a calcium ion exchange capacity of 211 mgCaCO_3_/g and a magnesium ion exchange capacity of 115 mgMgCO_3_/g using kaolin as the raw material. Low silicon X-type zeolite (LSX) with a SiO_2_/Al_2_O_3_ ratio of 2.0~2.2 has a large exchangeable cation capacity because its skeleton Si/Al ratio is close to 1, and it has attracted wide attention as a detergent builder for its high adsorption capacity of calcium ions and magnesium ions with large ionic radius. Kuhl et al. [27] synthesized LSX zeolite from chemical raw materials and found that the magnesium ion exchange capacity of LSX zeolite was 3 times that of 4A zeolite within 10 min. When the exchange time was 1 min, the amount of LSX zeolite was only 60% of 4A zeolite under the same conditions of calcium ion removal. However, the manufacturing cost of synthetic LSX zeolite from chemical agents is high [28], so its use as a detergent builder is limited.

Currently, the synthesis of LSX zeolites from rice husk ash, fly ash, potassium feldspar, coal gangue, kaolin, and lithium slag has been reported. Tontisirin et al. [29] used a hydrothermal method to synthesize LSX zeolite (SiO_2_/Al_2_O_3_ ratio of 2.18) from rice husk ash supplemented with aluminum and NaAlO_2_. The sample had trace type A zeolite heterophase with a BET-specific surface area of 499 m^2^/g and a grain size of 3~6 μm. Adamczyk et al. [30] synthesized LSX zeolite (SiO_2_/Al_2_O_3_ ratio of 2.06) with fly ash. The sample contained 41 wt% X zeolite with a small amount of unreacted raw materials and soalite. Han et al. [31] used potash feldspar to synthesize LSX zeolite, including trace A-type zeolite heterophase with a grain size of 5–8 μm. The BET-specific surface area of the zeolite sample was 467.8 m^2^/g. Liu et al. [32] synthesized pure-phase LSX zeolite (SiO_2_/Al_2_O_3_ ratio of 2.10) from coal gangue with a grain size of 5–8 μm and a BET-specific surface area of 634 m^2^/g. Basaldella et al. [33] synthesized a pure-phase LSX zeolite with a grain size of 4~5 μm using kaolin as the raw material. Outram et al. [34] used lithium slag as the raw material to synthesize LSX zeolite (SiO_2_/Al_2_O_3_ ratio of 2.2) with 63.7 wt% X zeolite and quartz, sodalite, and other heterophase. LSX zeolites synthesized from the above-mentioned industrial wastes are mainly used for removing heavy metal ions in the solution [35] and pressure swing adsorption separation [32,36]. There is no report on the performance of preparing LSX zeolite from waste residue used for the detergent builder.

In summary, the purity and specific surface area of LSX zeolite prepared from waste residue were poor. So far, there have been no reports on the synthesis of pure-phase LSX zeolite from lithium slag. There is also no report on the ion exchange capacity and ion exchange rate of calcium and magnesium ions as the detergent builder of LSX zeolite from waste residue. Therefore, the hydrothermal synthesis process of pure-phase KNaLSX zeolite from lithium slag was studied using a suitable sodium hydroxide to potassium hydroxide ratio, crystallization temperature, and crystallization time. The phase, chemical composition, morphology, thermal stability, functional group, and pore structure of the synthesized KNaLSX zeolite were characterized by XRD, XRF, SEM, TG-DTA, FT-IR, and N_2_ adsorption measurement. The ion exchange capacity and the ion exchange rate curves of calcium and magnesium ions within 15 min on KNaLSX zeolite were determined, and the competitive exchange relationship of calcium and magnesium ions on KNaLSX zeolite was investigated. The study aims to prepare pure-phase LSX from lithium slag and to provide the basis for developing a fast-washing detergent builder.

## 2. Experimental

### 2.1. Raw Materials and Main Reagents

Lithium slag was provided by a lithium company, and the phase structure and element compositions are shown in Figure S1 and Table S1. Anhydrous calcium chloride (≥96%, AR) was purchased from Sinopharm Chemical Reagent Co., Ltd., Shanghai, China; magnesium chloride hexahydrate (≥98%, AR) was purchased from Xilong Chemical Co., Ltd., Guangdong, China; commercial KNaLSX zeolite (named as KNaLSX-C) was purchased from Alfa Esha (China) Chemical Co., Ltd., Beijing, China; commercial 4A zeolite was purchased from Shandong Lanzhirun Environmental Protection Technology Co., Ltd., Weifang, China.

### 2.2. Synthesis of KNaLSX Zeolite from Lithium Slag

**Figure 1:**
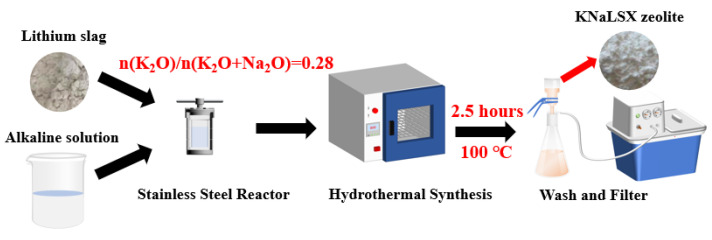
The diagram of the hydrothermal synthesis process of KNaLSX zeolite from lithium slag.

The diagram of the hydrothermal synthesis of KNaLSX zeolite from lithium slag is shown in Figure 1. In a stainless steel reactor lined with polytetrafluoroethylene, 10 g of lithium slag and 133 mL of NaOH and KOH mixed solution were added and then mixed evenly. The mixture was placed in an oven and then crystallized at 90~110 °C for 0.5~3 h. The solution was then cooled and filtered, and the solid sample was washed and dried to obtain a powder sample (named “KNaLSX-X-Y-Z”), where X represented the potassium/alkali ratio, Y represented the crystallization temperature, and Z represented the crystallization time.

### 2.3. Characterization of Synthetic Sample

The phases of KNaLSX were determined by XRD (Smartlab^TM^ 9KW X-ray diffractometer, Rigaku Corporation, Tokyo, Japan). The chemical compositions of KNaLSX were measured by XRF (ZSX Primus Ⅱ X-ray fluorescence spectrometer, Rigaku Corporation, Tokyo, Japan). The morphology of KNaLSX was scanned by SEM (S-4800 field emission scanning electron microscope, Hitachi Corporation, Tokyo, Japan). The TG-DTA curves of KNaLSX were measured by the WCT-1 thermogravimetric analyzer (Beijing Optical Instrument Factory Co., Ltd., Beijing, China). The surface functional group information of KNaLSX was measured by the Avatar-360 infrared spectrometer (Nicole Instruments Inc., Lanoka Harbor, NJ, USA). The specific surface area and pore structure of KNaLSX were determined from the measured N_2_ adsorption equilibrium data by the Belsorp Max physical adsorption instrument (Microtrac BEL Corp., Osaka, Japan).

### 2.4. Determination of Ion Exchange Capacity and Ion Exchange Rate Curves of Calcium and Magnesium Ions

#### 2.4.1. Ion Exchange Capacity of Calcium and Magnesium Ions

The calcium ion exchange capacity of KNaLSX and 4A zeolites was determined with QB/T 1768-2003 [37]. The magnesium ion exchange capacity of KNaLSX and 4A zeolites was determined with QB/T 19421-2003 [38].

#### 2.4.2. Ion Exchange Rate Curve of Calcium and Magnesium Ions

First, 500 mL of 0.005 mol/L calcium chloride/magnesium chloride solution was added into a three-neck flask, and the pH value of the solution was then adjusted to 10.5 ± 0.1, 0.5 g KNaLSX sample was then added with a stirring speed of 600 rpm at 35 °C for the ion exchange of calcium or magnesium. The liquid samples were taken every 20 s within 0~3 min, and then every 3 min from 3 to 15 min. The concentrations of calcium and magnesium ions in solution at different exchange times were determined by ICP (AVIO-200 Inductively Coupled Plasma Spectrometer, Perkinelmer Inc., US). The test conditions were that the plasma gas (Ar) flow rate was 14 L/min with the auxiliary gas (Ar) flow rate of 0.4 L/min, atomizer gas (Ar) flow rate was 0.7 L/min, and RF power was 1300 W.

According to the concentration changes of calcium and magnesium ions in the solution before and after ion exchange, the ion exchange capacity of calcium and magnesium ions (*q_t_*) on KNaLSX and 4A zeolite under different ion exchange times (*t*) is calculated by Equation (1), as follows:

(1) $$q_{t}=\frac{(C_{0}\;-C_{t})\times V\times M_{1}}{m\times (1-X)\times M_{2}}$$

where *C_0_* is the initial concentration of calcium/magnesium ions (g/L), *C_t_* is the concentration of calcium/magnesium ions at time t in the solution (g/L), *V* is the solution volume (L), *m* is the amount of zeolite (g), *M*_1_ is the molar mass of calcium carbonate/magnesium carbonate (g/mol), *M*_2_ is the molar mass of calcium/magnesium ion (g/mol), and *X* is the ignition loss mass of the zeolite (%).

The pseudo-first-order kinetic model (PFO model) and pseudo-second-order kinetic model (PSO model) were used to fit the ion change in exchange capacity of KNaLSX and 4A zeolites for calcium and magnesium ions with time t. The equations of PFO and PSO models are shown in Equations (2) and (3), respectively.

(2) $$Q_{t}=q_{e}\left(1-e^{{-k}_{1}t}\right)$$

(3) $$q_{t}=\frac{{q_{e}}^{2}k_{2}t}{1+q_{e}k_{2}t}$$

where, *k*_1_ (1/min) is the adsorption rate constant of the PFO kinetic model, *k*_2_ (g/(mg·min)) is the adsorption rate constant of the PSO kinetic model, *q_e_* (g/mg) is the theoretical equilibrium ion exchange capacity of calcium and magnesium ions, *q_t_* (g/mg) is the ion exchange capacity of calcium and magnesium ions at the ion exchange time *t*, and *t* (min) is the ion exchange time.

## 3. Results and Discussions

### 3.1. Preparation of KNaLSX Zeolite from Lithium Slag

Based on the synthesis of NaX [11,16,17] and NaA/NaX co-crystalline zeolite [12,13] from lithium slag from previous studies, KNaLSX zeolite was prepared by the hydrothermal synthesis method. Effects of sodium hydroxide to potassium hydroxide ratio, crystallization temperature, and crystallization time on the phase and relative crystallinity of KNaLSX zeolite were investigated, and the preparation conditions were optimized. The relative crystallinity was calculated from the peak area of the characteristic peak of the synthesis sample; the equation is shown in Equation (4), as follows:

(4) $$\mathrm{Relative}\;\mathrm{crystallinity}=\frac{\mathrm{peak}\;\mathrm{area}\;\mathrm{of}\;\mathrm{sample}}{\mathrm{peak}\;\mathrm{area}\;\mathrm{of}\;\mathrm{standard}\;\mathrm{sample}}\times 100\%$$

#### 3.1.1. Potassium to Alkali Ratio

**Figure 2:**
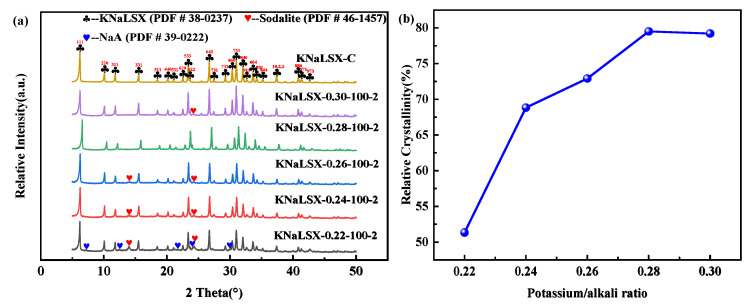
Synthetic samples at different potassium/alkali ratios: (**a**) XRD patterns and (**b**) relative crystallinity.

Potassium ion was an accelerant for the formation of double six-membered rings (D6R, Si/Al = 1), and D6R was one of the secondary structural units of zeolite X [39]. The proportion of potassium in base ions is closely related to the selective formation of KNaLSX. The effects of the sodium hydroxide to potassium hydroxide ratio (n(K_2_O)/n(K_2_O + Na_2_O)) between 0.22 and 0.30 on the phase and relative crystallinity of KNaLSX zeolites synthesized from lithium slag were investigated, and the results are shown in Figure 2.

As can be seen from Figure 2, when the potassium/alkali ratio is 0.22, NaA zeolite and hydroxysodalite appear in the synthetic sample. The relative crystallinity of the KNaLSX zeolite is only 51.33%. This is due to the low content of potassium ions in the synthetic system, which makes it difficult for the four-member rings (4R) formed by silicaluminate to form double six-member rings (D6R). The high content of four-member rings in the system is conducive to the growth of NaA zeolite and hydroxysodalite. When the potassium/alkali ratio increases to 0.24~0.26, only a small amount of hydroxysodalite appears in the sample, the peaks for NaA zeolite disappear, and the relative crystallinity of KNaLSX zeolite also increases to 68.86~72.91%. By increasing the potassium/alkali ratio to 0.28~0.30, the relative crystallinity is 79.51% and 79.21%, respectively. The content of potassium ions increases in the solution, which is favorable for the formation of D6R rather than 4R, thus inhibiting the growth of NaA zeolite. Therefore, the suitable potassium-alkali ratio of KNaLSX zeolite synthesized from lithium slag is 0.28.

#### 3.1.2. Crystallization Temperature

**Figure 3:**
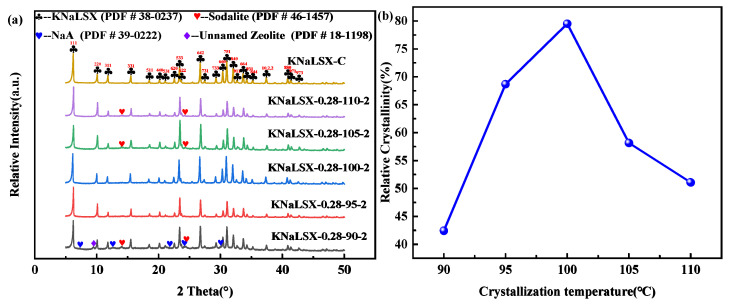
Synthetic samples at different crystallization temperatures: (**a**) XRD patterns and (**b**) relative crystallinity.

The effect of crystallization temperature between 90 °C and 110 °C on the phase and relative crystallinity of the synthesized KNaLSX zeolites was investigated, and the results are shown in Figure 3.

As can be seen from Figure 3, when the crystallization temperature is 90 °C, there are hydroxysodalite, NaA, and unnamed zeolites in the synthesized sample because of the low crystallization temperature. The relative crystallinity of KNaLSX zeolite is only 42.43%, due to the slow dissolution rate of Si and Al in the lithium slag, which leads to the slow growth rate of the zeolite. With the increase in temperature from 95 °C to 100 °C, the maximum relative crystallinity of KNaLSX zeolite reaches 79.51% because the increase in crystallization temperature accelerates the growth of crystal nuclei and the relative crystallinity of the zeolite increases. The relative crystallinity of KNaLSX decreases to 58.16% and 51.09% with the increase in crystallization temperature to 105 °C and 110 °C, and hydroxysodalite heterophase appears. The structure of the samples tends to be more stable. Because the 4R structures in hydroxysodalite are more stable than those in the 6R and D6R in the KNaLSX zeolite, some KNaLSX zeolites are converted to hydroxysodalite. Therefore, the suitable crystallization temperature of KNaLSX zeolite synthesized from lithium slag is 100 °C.

#### 3.1.3. Crystallization Time

**Figure 4:**
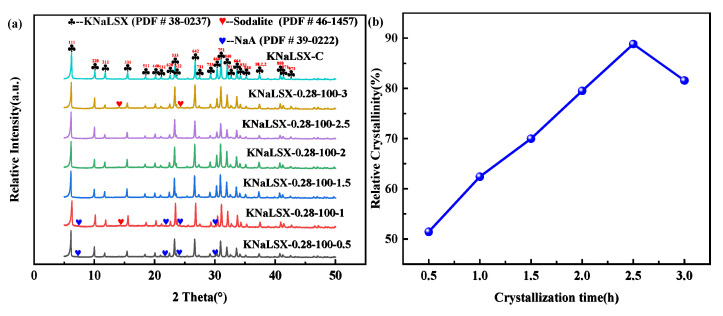
Synthetic samples at different crystallization times: (**a**) XRD patterns and (**b**) relative crystallinity.

The effects of crystallization time between 0.5 h and 3 h on the phase and relative crystallinity of the synthesized KNaLSX zeolites were investigated, and the results are shown in Figure 4.

As can be seen from Figure 4, NaA zeolite appears in the synthetic samples when the crystallization time is between 0.5 and 1 h. When the crystallization time is 2.5 h, the relative crystallinity of KNaLSX zeolite is up to 88.83%. The relative crystallinity of the zeolite decreases to 81.58% when the crystallization time extends to 3 h. NaA zeolite can be converted into KNaLSX with the proper extension of crystallization time, and the crystallinity increases. However, when the crystallization time is long enough, the concentration of Si^4+^ and Al^3+^ in the synthesis system changes, resulting in the formation of a dense structure of hydroxysodalite by the combination of the 4R with the SOD cage. Moreover, some KNaLSX is transformed into sodalite, and the crystallinity is reduced. Therefore, the suitable crystallization time of KNaLSX zeolite synthesized from lithium slag is 2.5 h.

In summary, the pure-phase KNaLSX zeolite can be successfully synthesized from lithium slag without the addition of silica or aluminum sources under the conditions of a potassium/alkali ratio of 0.28, a crystallization temperature of 100 °C, and a crystallization time of 2.5 h. The relative crystallinity is 88.83%. So far, there has been no report on the synthesis of KNaLSX zeolite with high relative crystallinity (this work) using lithium slag.

### 3.2. Characterization of the Synthesized KNaLSX

KNaLSX-0.28-100-2.5 (hereinafter referred to as KNaLSX) was characterized by SEM, XRF, TG-DTA, and nitrogen adsorption/desorption, and the results were compared with the standard sample (KNALSX-C).

#### 3.2.1. Crystal Morphology

**Figure 5:**
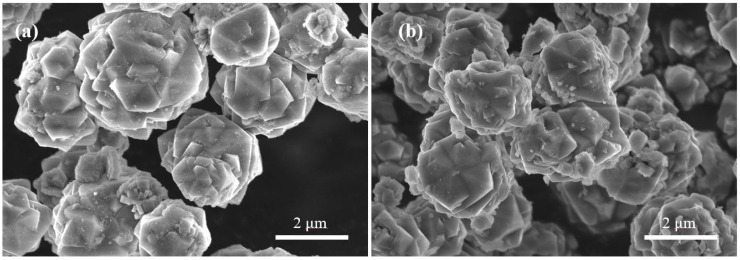
SEM images of (**a**) KNaLSX and (**b**) KNaLSX-C.

SEM images of KNaLSX and KNaLSX-C are shown in Figure 5.

As can be seen from Figure 5, KNaLSX and KNALSX-C zeolites have typical octahedral structures with uniform particle sizes of about 3~4 μm. Both KNaLSX and KNaLSX-C grains have agglomeration. The continuous dissolution of silicon and aluminum in the lithium slag promotes the formation of a large number of X zeolite crystal nuclei on the gel surface and rapid growth. The continuous growth of secondary structural units and cage structures, and the appearance of a large number of twins lead to the agglomeration of grains.

#### 3.2.2. Chemical Composition

The chemical compositions of KNaLSX and KNaLSX-C are shown in Table 1.

It can be seen from Table 1 that the contents of SiO_2_ and Al_2_O_3_ in KNaLSX zeolite are 41.55% and 35.12%, respectively, and SiO_2_/Al_2_O_3_ of KNaLSX is 2.01, which is a low silica-aluminum ratio zeolite and similar to that of KNaLSX-C (2.0). The content of Na_2_O and K_2_O in KNaLSX is 13.82% and 8.46%, respectively, which is lower than that in KNaLSX-C (14.48% and 9.11%). This is because each aluminum oxide tetrahedron on the zeolite skeleton has a negative charge, and corresponding cations are needed to balance the charge. The molar ratio of SiO_2_/Al_2_O_3_ in KNaLSX is slightly higher than that of KNaLSX-C, which leads to the relatively low proportion of aluminum oxide tetrahedrons on the skeleton.

It can be seen that KNaLSX zeolite with a SiO_2_/Al_2_O_3_ ratio (n (SiO_2/_Al_2_O_3_)) of 2.01 is successfully synthesized using lithium slag.

#### 3.2.3. Thermal Stability

**Figure 6:**
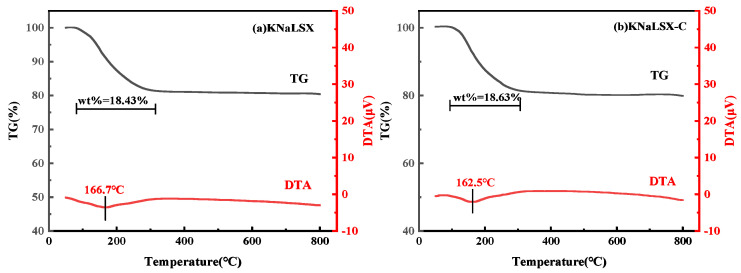
TG-DTA curves of (**a**) KNaLSX and (**b**) KNaLSX-C.

The TG-DTA curves of KNaLSX and KNaLSX-C were determined, and the results are shown in Figure 6.

As can be seen from Figure 6, the TG curves of KNaLSX and KNalSX-C are the same, and both have significant weight loss before 300 °C. The weight losses of KNaLSX and KNaLSX-C are 18.43% and 18.63%, respectively. On the DTA curve, the heat absorption peaks of KNaLSX and KNALSX-C appear at 166.7 °C and 162.5 °C, respectively, which are generated by removing water from the surface and pore channels of the zeolite. When the temperature increases to 800 °C, neither KNaLSX nor KNaLSX-C zeolites show obvious weight loss, indicating that the synthetic KNaLSX is thermally stable.

#### 3.2.4. Specific Surface Area and Pore Structure

**Figure 7:**
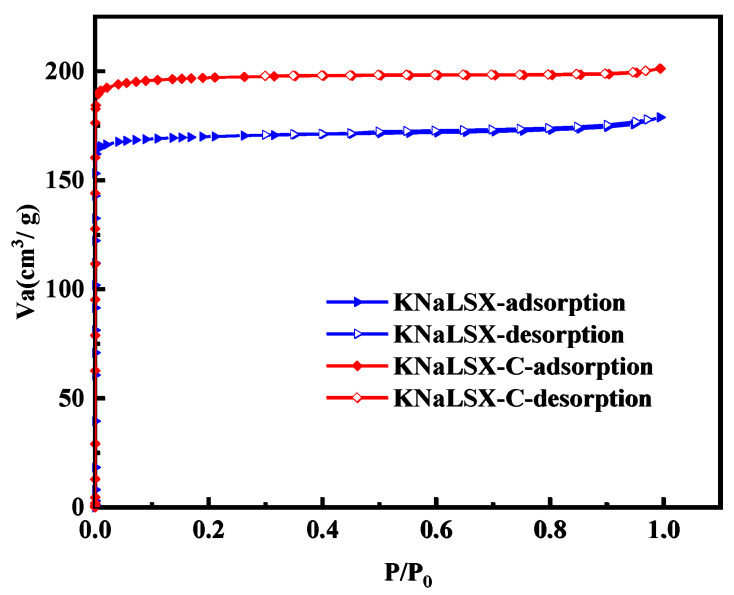
N_2_ adsorption/desorption isotherms of KNaLSX and KNaLSX-C.

**Figure 8:**
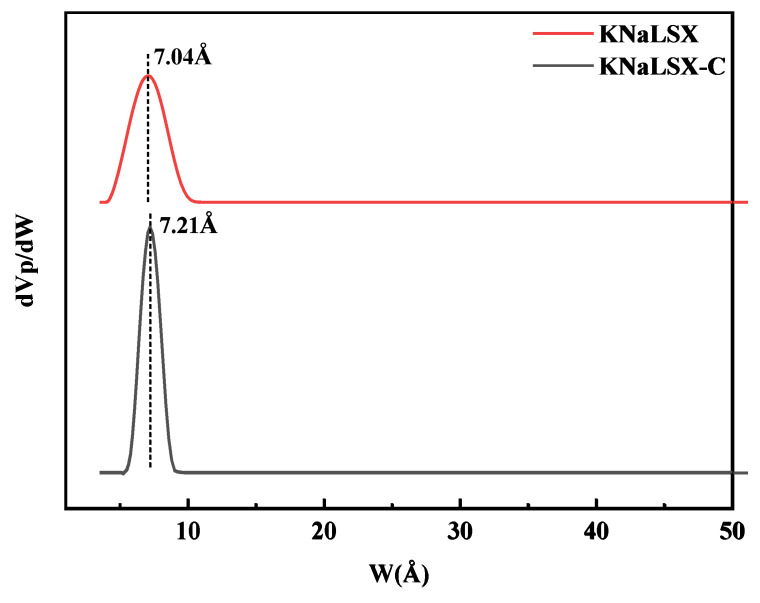
Pore size distribution of KNaLSX and KNaLSX-C calculated by the NLDFT method.

The adsorption/desorption isotherms of nitrogen on KNaLSX and KNaLSX-C at 77 K were measured, and the results are shown in Figure 7. The Non-local Density Functional Theory (NLDFT) model was used to calculate the pore size distribution, the BET model was used to calculate the specific surface area, and the t-plot method was used to calculate the micropore volume. The results are shown in Figure 8 and Table 2.

As can be seen from Figure 7, nitrogen adsorption/desorption isotherms on KNaLSX and KNaLSX-C are both type I isotherms. The nitrogen adsorption capacity of KNaLSX zeolite increases sharply with an increase in relative partial pressure (P/P_0_) to 0.04. When P/P_0_ is close to 0.1, the nitrogen adsorption capacity of KNaLSX zeolite is close to its saturation. The equilibrium adsorption capacity of nitrogen on KNaLSX and KNaLSX-C zeolites reaches 175 cm^3^/g and 199 cm^3^/g, respectively, and the nitrogen adsorption capacity of KNaLSX is 87.94% of KNaLSX-C. The pore size distribution is shown in Figure 8. The most probable pore size of synthetic KNaLSX is 7.04 Å, which is a little less than that of KNalSX-C (7.21 Å). At the same time, the number of KNALSX-C pores is larger than that of KNaLSX. As can be seen from Table 2, the BET-specific surface area, total pore volume, and micropore volume of KNaLSX are 715 m^2^/g, 0.264 cm^3^/g, and 0.264 cm^3^/g, respectively, which are about 86~87% of the commercial KNALSX-C zeolite.

The compared properties of KNaLSX synthesized from lithium slag with the properties of LSX zeolite synthesized from industrial waste listed in the literature, such as SiO_2_/Al_2_O_3_ ratio, particle size, and specific surface area, are shown in Table S1. The SiO_2_/Al_2_O_3_ ratio of pure-phase KNaLSX zeolite synthesized from lithium slag is 2.01 (the SiO_2_/Al_2_O_3_ ratio of LSX zeolite synthesized from industrial waste residue in the literature is >2.06), and the BET specific surface area is 715 m^2^/g, which is better than that reported in the literature (<640 m^2^/g). The particle size is uniform, and the particle size is 3~4 μm.

### 3.3. Ion Exchange Capacity and Ion Exchange Rate Performance of KNaLSX Zeolite for Calcium and Magnesium Ions

#### 3.3.1. Ion Exchange Capacity for Calcium and Magnesium Ions

According to the QB/T 1768-2003 and QB/T 19421-2003, under the conditions of 35 °C, solid-liquid ratio of 1 g/L, initial calcium concentration of 200 mg/L (initial magnesium concentration of 120 mg/L) and ion exchange time of 20 min, the ion exchange capacities of calcium and magnesium ions on KNaLSX, KNaLSX-C and 4A zeolite were determined respectively. The results are shown in Table 3.

As can be seen from Table 3, the calcium ion exchange capacity of KNaLSX, KNaLSX-C, and 4A zeolite is 302 mgCaCO_3_/g, 317 mgCaCO_3_/g, and 325 mgCaCO_3_/g, respectively. The ion exchange capacity of KNaLSX for calcium ions is 95.27% of KNaLSX-C, which is higher than the relative crystallinity (88.83%), but it is consistent with the ratio of the total content of Na^+^ and K^+^ in KNaLSX and KNaLSX-C (94.45%). This indicates that the calcium ion exchange capacity of KNaLSX zeolite is directly related to the monovalent cation content in the zeolite skeleton. According to the previous study [40], the selectivity of calcium ions is highest on type A zeolite and monotonically increases with the increase in Al content in the zeolite. The exchange capacity of KNaLSX for calcium ions is lower than that of 4A zeolite, but it meets the first-grade requirements of zeolite for a detergent builder (≥295 mgCaCO_3_/g). This is because the content of monovalent cation in the skeleton of 4A is higher than that of KNaLSX zeolite [41], which leads to a higher calcium ion exchange capacity.

Meanwhile, it can be seen from Table 3 that the magnesium ion exchange capacity of KNaLSX and KNaLSX-C is 191 mgMgCO_3_/g and 202 mgMgCO_3_/g, respectively, which is twice that of 4A zeolite (92 mgMgCO_3_/g). This is because magnesium ion exchange adsorption is not only related to the micropore size of the zeolite but also to the charge distribution and location of the cation (Na^+^, K^+^) in the zeolite. The ion exchange capacity of magnesium ion is determined by its kinetic diameter, the pore size of the zeolite, the local electric field in the crystal, the interaction force between magnesium ion and the zeolite skeleton, and the diffusion process of magnesium ion in the pores. The charge density of magnesium ions is higher than that of calcium ions, and the diameter of hydrated magnesium ions is larger than that of hydrated calcium ions [42]. The pore diameter of 4A zeolite is only 0.38 nm, and the diffusion resistance of magnesium ions into the pore channel of 4A zeolite is high. Magnesium ion is preferentially exchanged with Na^+^ on the surface or orifice, and the ion exchange capacity of 4A zeolite for magnesium ions is poor.

To compare the calcium and magnesium ion exchange performance of KNaLSX zeolite, the calcium and magnesium ion exchange performance of 4A and NaX zeolites synthesized from industrial waste residue in the literature was compared, and the results are shown in Table 4.

As can be seen from Table 4, the calcium ion exchange capacity of synthetic KNaLSX zeolite is 302 mgCaCO_3_/g, which is significantly higher than that of NaX zeolite synthesized from other industrial waste residue [23,24,25,26] (150–211 mgCaCO_3_/g). The ion exchange capacity of calcium ions is similar to that of the 4A zeolite synthesized from industrial waste residue [43,44,45,46] (142.5–318.5 mgCaCO_3_/g), which further indicates that the crystallinity of the KNaLSX zeolite synthesized from lithium slag is higher and the silica-aluminum ratio is closer to that of the A-type zeolite. The ion exchange capacity of KNaLSX zeolite for magnesium ion is 191 mgMgCO_3_/g, which is 1.6 times that of X-type zeolite synthesized from kaolin [26] under the same test conditions.

It can be seen that the calcium ion exchange capacity of synthetic KNaLSX zeolite in the paper has reached the requirement of the first-grade for detergent builder (≥295 mgCaCO_3_/g), and the ion exchange capacity of magnesium ion is 1.6 times than that of NaX zeolite synthesized by kaolin. So KNaLSX is suitable to be used as a detergent builder.

#### 3.3.2. Ion Exchange Rate for Calcium and Magnesium Ions of KNaLSX

**Figure 9:**
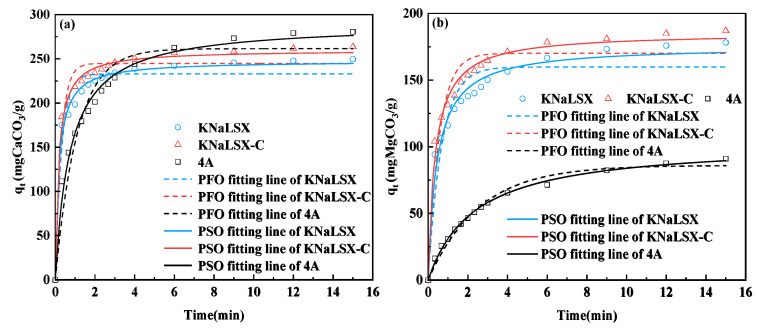
(**a**) Calcium ion and (**b**) magnesium ion exchange rate curves of the zeolites and their kinetic model fitting.

In order to investigate the ion exchange rate of calcium and magnesium ions on KNaLSX zeolite, the ion exchange rate curves of KNaLSX, KNaLSX-C, and commercial 4A zeolite for calcium and magnesium ions were determined (within 15 min) under the conditions of 30 °C, a solid-liquid ratio of 1 g/L, and a 200 mg/L calcium ion concentration (120 mg/L magnesium ion concentration). Pseudo-first-order (PFO) and pseudo-second-order (PSO) adsorption kinetics models were used to fit the rate curves of calcium and magnesium ions. The results are shown in Figure 9 and Table 5.

As can be seen from Figure 9a, in the initial stage of ion exchange, the ion exchange rate of calcium ions in KNaLSX and KNaLSX-C zeolite is greater than 4A and reaches ion exchange equilibrium within 3 min, which is only 1/5 of that in 4A zeolite (15 min). This is because the pore size of KNaLSX zeolite (~7.2 Å) is larger than that of 4A zeolite (~4 Å), and the larger the pore size of KNaLSX zeolite is, the faster the ion exchange of calcium ions [27].

As can be seen from Figure 9b, the ion exchange equilibrium time of magnesium ions on KNaLSX and KNaLSX-C zeolites is 6 min. Compared with calcium ions, the ion exchange equilibrium time of magnesium ions is longer, mainly because magnesium ions exist in their hydrated form in an aqueous solution, and the larger ion radius affects their ion exchange rate. However, under the same conditions, 4A zeolite cannot reach the adsorption equilibrium for magnesium ions within 15 min, indicating that the rate of KNaLSX and KNaLSX-C zeolites for magnesium ions is significantly faster than that of 4A zeolite.

As can be seen from Table 5, the fitting degree of KNaLSX, KNaLSX-C, and 4A zeolites for calcium and magnesium ion exchange rate curves by the pseudo-first-order kinetic model (PFO) is low (0.90~0.95), while the fitting degree of the pseudo-second-order kinetic model (PSO) is greater than 0.98. Thus, the pseudo-second-order kinetic model can be better used to describe the ion exchange process of calcium and magnesium ions on KNaLSX, KNaLSX-C, and 4A zeolite. Using the PSO model, the theoretical equilibrium ion exchange capacity *q_e_* values of KNaLSX, KNaLSX-C, and 4A zeolites for calcium and magnesium ions are consistent with the experimental values. The ion exchange rate constant (*k_2_*) of KNaLSX for calcium ions is 0.0221 g/(mg·min), which is similar to KNaLSX-C and about 5 times that of 4A zeolite 0.0046 g/(mg·min). The ion exchange rate constant of KNaLSX and KNaLSX-C zeolites for magnesium ions is also 3 times that of 4A zeolite.

In summary, from the ion exchange rate, KNaLSX zeolite has almost the same ion exchange rate for calcium and magnesium ions, but the exchange rate constant is 5 times and 3 times that of 4A zeolite, respectively, indicating that KNaLSX zeolite can be used to develop a detergent builder for rapid washing.

#### 3.3.3. Competitive Ion Exchange Relationship of Calcium and Magnesium Ions on Zeolites

**Figure 10:**
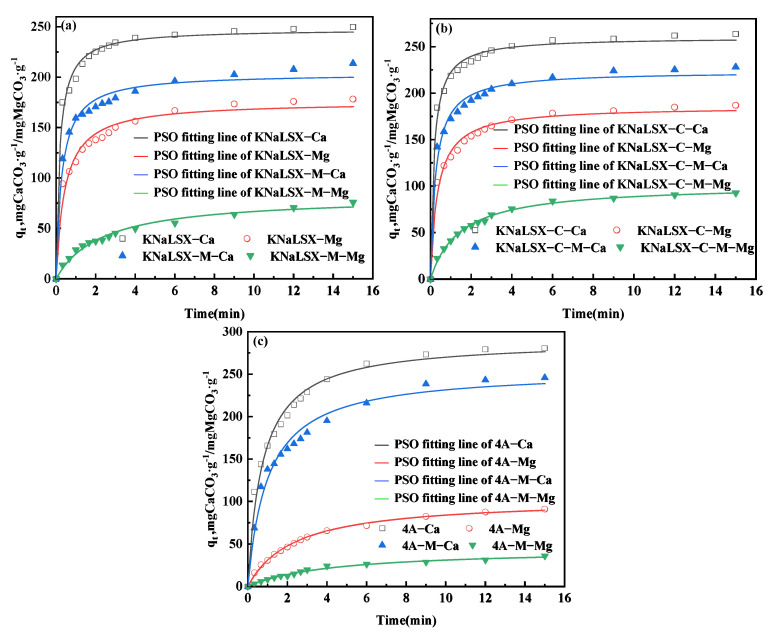
The ion exchange rate curves and kinetic model fitting of the mixed solution of calcium and magnesium ions by (**a**) KNaLSX, (**b**) KNaLSX-C, and (**c**) 4A zeolites.

In order to further explore the adsorption mechanism and competitive adsorption relationship of calcium and magnesium ions on zeolites, under the conditions of 30 °C, a solid-liquid ratio of 1 g/L, a calcium ion concentration of 200 mg/L, and a magnesium ion concentration of 120 mg/L, the ion exchange rate curves of calcium and magnesium ions in the mixed solution on KNaLSX, KNaLSX-C, and commercial 4A zeolite were determined, respectively, within 15 min. A pseudo-second-order (PSO) adsorption kinetic model was used to fit the rate curves of calcium and magnesium ions, respectively, and the results are shown in Figure 10 and Table 6.

As can be seen from Figure 10, compared with a single solution of calcium and magnesium ions, the shape of the ion exchange rate curves of calcium and magnesium ions by KNaLSX, KNaLSX-C, and 4A zeolites in the mixed solution of calcium and magnesium ions does not change significantly, but the ion exchange equilibrium capacity of calcium and magnesium ions decreases significantly. Taking the KNaLSX as an example, the ion exchange equilibrium capacities of calcium and magnesium ions in the mixed solution are 213 mgCaCO_3_/g and 75 mgMgCO_3_/g, respectively, which are only 85.5% and 43.6% of those in the single solution. The effect of calcium ions on the ion exchange process of a mixed solution is obviously smaller than that of magnesium ions. This is because the ion exchange rate of calcium ions is faster, and it can preferentially occupy the adsorption site, so the ion exchange capacity of magnesium ions is greatly reduced, which is the same as the previous study [21].

It can be seen from Table 5 and Table 6 that KNaLSX and KNaLSX-C significantly reduce the ion exchange rate constants of calcium and magnesium ions in a mixed solution of calcium and magnesium ions compared with a single solution. Taking KNaLSX as an example, the exchange rate constants (*k*_2_) of calcium and magnesium ions in the mixed solution are 0.0168 g/(mg·min) and 0.0052 g/(mg·min), respectively, which decrease by 24% and 59% of that compared with the single solution and have a greater impact on the ion exchange rate of magnesium ions. However, the ion exchange rate constant of 4A zeolite for calcium and magnesium ions in the mixed solution is almost the same as that in a single solution, which may be due to the slow ion exchange rate of 4A zeolite for calcium and magnesium ions [21], and the addition of other ions in a single solution has little effect on the ion exchange diffusion process.

In conclusion, in the mixed solution, the KNaLSX zeolite has a decrease in the ion exchange capacity and rate of calcium and magnesium ions and has a greater effect on magnesium ions. However, the exchange rate of KNaLSX zeolite for calcium ion and magnesium ion is still 4 times and 2 times that of 4A zeolite, respectively.

## 4. Conclusions

The pure-phase KNaLSX zeolite is successfully synthesized from the lithium slag without adding silicon or aluminum sources, and it has a SiO_2_/Al_2_O_3_ ratio of 2.01 with a uniform grain size of 3~4 μm. The ion exchange capacity of the synthesized KNaLSX zeolite for calcium ions is 302 mgCaCO_3_/g, which meets the requirement of first-grade zeolite for a detergent builder (≥295 mgCaCO_3_/g). The ion exchange capacity of KNaLSX for magnesium ions is 2 times that of 4A zeolite. Under the same test conditions, the ion exchange rate constants of calcium and magnesium ions are 5 times and 3 times that of 4A zeolite, respectively.

In this work, the pure-phase low silicon X-type zeolite (LSX) is prepared by lithium slag, which has a large capacity for magnesium ion exchange and a fast exchange rate of calcium and magnesium ions. Using KNaLSX as a detergent builder is helpful to improve the water-softening property and reduce the cost of a detergent builder. The paper results provide a basis for high-value resource utilization of lithium slag and the development of a detergent builder for rapid washing.

## Figures and Tables

**Table 1:** Chemical compositions of synthetic KNaLSX and KNaLSX-C zeolite (wt%).

Component	SiO_2_	Al_2_O_3_	Na_2_O	K_2_O	CaO	Fe_2_O_3_	n (SiO_2_/Al_2_O_3_)
KNaLSX	41.55	35.12	13.82	8.46	0.55	0.13	2.01
KNaLSX-C	41.19	35.10	14.48	9.11	0.03	0.02	2.00

**Table 2:** Specific surface area and pore-structure parameters of KNaLSX and KNaLSX-C.

Sample	BET Specific Surface Area (m^2^/g)	Total Pore Volume (cm^3^/g)	Micropore Volume (cm^3^/g)
KNaLSX	715	0.238	0.235
KNaLSX-C	827	0.264	0.261

**Table 3:** Ion exchange capacity of calcium and magnesium ions of KNaLSX, KNaLSX-C, and 4A zeolites.

Sample	KNaLSX	KNaLSX-C	4A
Calcium exchange capacity (mgCaCO_3_/g)	302	317	325
Magnesium exchange capacity (mgMgCO_3_/g)	191	202	92

**Table 4:** Exchange capacity and exchange rate of calcium and magnesium ions in different zeolites.

Zeolite Type	Raw Material	Calcium Exchange Capacity (mgCaCO_3_/g)	Magnesium Exchange Capacity (mgMgCO_3_/g)	Reference
4A	Fly ash	142.5~188.5	——	[43]
	Loess	318.5	——	[44]
	Kaolin	310	——	[45]
	Rice husk ash	272.5	——	[46]
NaX	Lithium slag	150	——	[23]
	Fly ash	119.5	119	[24]
	Gold mine Tailing	62.78	12.56	[25]
	Kaolin	211	115	[26]
LSX	Lithium slag	302	191	This work

**Table 5:** Fitting parameters of different kinetic models of calcium and magnesium ion exchange on KNaLSX, KNaLSX-C, and 4A zeolites.

Model	Sample	Constant	KNaLSX	KNaLSX-C	4A
PFO	Calcium ion	*q_e_*, mg/g	232.9	244.8	261.5
		*k*_1_, 1/min	3.0978	3.3477	0.9022
		R^2^	0.95	0.96	0.94
	Magnesium ion	*q_e_*, mg/g	159.9	170.1	86.1
		*k*_1_, 1/min	1.456	1.7752	0.3934
		R^2^	0.90	0.92	0.95
PSO	Calcium ion	*q_e_*, mg/g	247.7	256.0	290.3
		*k*_2_, g/(mg·min)	0.0221	0.0227	0.0046
		R^2^	0.99	0.99	0.99
	Magnesium ion	*q_e_*, mg/g	175.8	185.5	103.6
		*k*_2_, g/(mg·min)	0.0128	0.0152	0.0041
		R^2^	0.98	0.99	0.99

**Table 6:** Fitting parameters of the PSO model of calcium and magnesium ion exchange on KNaLSX, KNaLSX-C, and 4A zeolites.

Model	Sample	Constant	KNaLSX-M	KNaLSX-C-M	4A-M
PSO	Calcium ion	*q_e_*, mg/g	203.7	223.5	255.1
		*k*_2_, g/(mg·min)	0.0168	0.0173	0.0039
		R^2^	0.98	0.99	0.98
	Magnesium ion	*q_e_*, mg/g	82.3	101.7	44.5
		*k*_2_, g/(mg·min)	0.0052	0.0067	0.0052
		R^2^	0.98	0.99	0.99

## Data Availability

Data is available on request from the authors.

## Supplementary Materials

The following supporting information can be downloaded at: https://www.mdpi.com/article/10.3390/ma17133181/s1, Figure S1: XRD pattern of lithium slag. Table S1: The chemical compositions and water content of different batches of lithium slag. Figure S2: SEM images of KNaLSX samples at different potassium/alkali ratios (a) 0.22 (b) 0.24 (c) 0.26 (d) 0.28 (e) 0.30. Figure S3: SEM images of KNaLSX samples at different crystallization temperatures (a) 90°C (b) 95°C (c) 100°C (d) 105°C (e) 110°C. Figure S4: SEM images of KNaLSX samples at different crystallization times (a) 0.5 h (b) 1 h (c) 1.5 h (d) 2 h (e) 2.5 h (f) 3 h. Figure S5: FT-IR patterns of KNaLSX samples at different crystallization times. Table S2: Properties of LSX zeolites synthesized from industrial waste. Refs [47,48,49,50,51].
