# Supplementary material for: Synthesis of Low-Silicon X-Type Zeolite from Lithium Slag and Its Fast Exchange Performance of Calcium and Magnesium Ions
Source: Materials (Basel). 2024 Jun 28;17(13):3181. doi: 10.3390/ma17133181 (PMC11242566; doi:10.3390/ma17133181)
Supplement: Supplementary file 1 [file materials-17-03181-s001.zip › materials-3065364-supplementary.pdf]

Supporting Information

Synthesis of low silicon X-type zeolite from lithium slag and its fast exchange performance of calcium and magnesium ions

Yu Wang, Longbin Deng, Lin Zhang, Qun Cui* and Haiyan Wang

College of Chemical Engineering, Nanjing Tech University, Nanjing, 211816, China

* Corresponding author: cuiqun@njtech.edu.cn. Tel: +86 025 83587188.

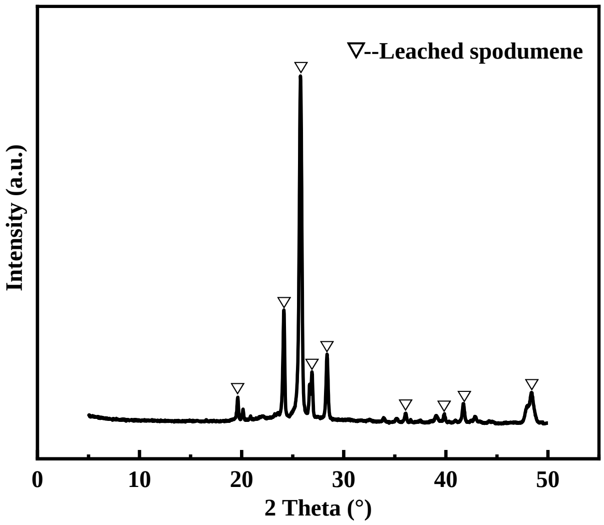

Figure S1 XRD pattern of lithium slag

Table S1 The chemical compositions and water content of different batches of lithium slag

Batches	Water content (wt%)	Chemical compositions without water (wt%)							
		SiO_2	Al_2O_3	Fe_2O_3	SO_3	Na_2O	K_2O	CaO	MgO
1#	3.15	70.78	27.01	0.24	0.30	0.12	0.13	0.11	0.05
2#	3.07	70.73	27.06	0.23	0.32	0.11	0.14	0.10	0.06
3#	3.13	70.75	27.04	0.23	0.31	0.12	0.13	0.10	0.05

It can be seen from Figure S1 and Table S1 that the main characteristic peak in the XRD pattern of lithium slag is leached spodumene, without any other impurity peaks, the main components of lithium slag are SiO_2 and Al_2O_3, lithium slag has a low content of impurities such as iron and calcium, a single crystal structure, and no need for pre-treatment. It can be directly used as a raw material for synthesizing KNaLSX zeolite.

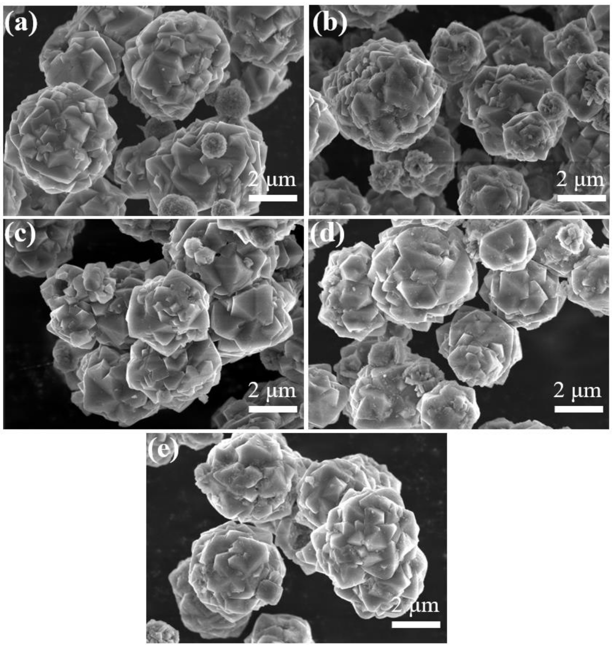

Figure S2. SEM images of KNaLSX samples at different potassium/alkali ratios

(a) 0.22 (b) 0.24 (c) 0.26 (d) 0.28 (e) 0.30

The **sy**nthetic samples with different potassium alkali ratios have typical octahedral structure with an average diameter of 3~4 μm. There are a few small grains in the sample with potassium alkali ratios between 0.22 and 0.26, which corresponds to sodalite heterophase in the phase. When potassium to alkali ratio increases to 0.28 and 0.30, the small grains disappear in the samples, which has the uniform particle size of 3~4 μm.

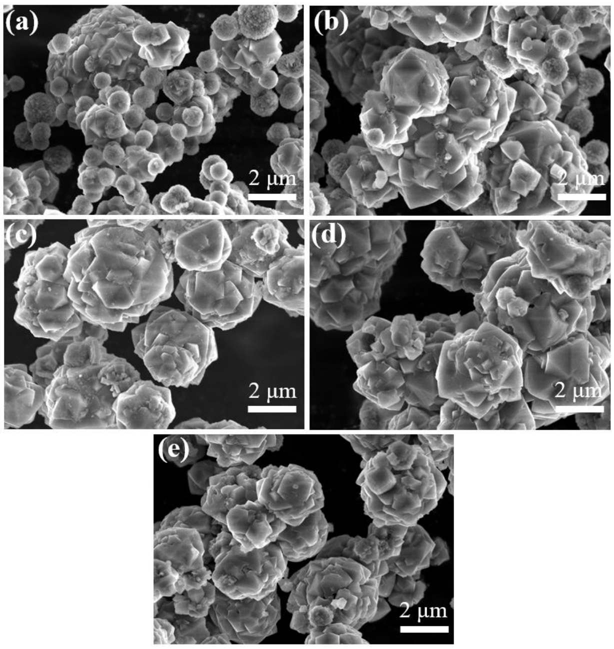

Figure S3. SEM images of KNaLSX samples at different crystallization temperatures (a) 90°C (b) 95°C (c) 100°C (d) 105°C (e) 110°C

The **sy**nthetic samples with different crystallization temperatures have typical octahedral structure with an average diameter of 3~4 μm. There are a large number of small grains in the samples at 90°C, which is due to the low crystallization temperature, slow growth and low crystallinity of the zeolite samples, and the presence of NaA and sodite heterophase. With the increase of crystallization temperature, the particle size increases and the crystallinity of the sample increases. When the temperature increases to 105 and 110°C, a small amount of small grains attach around the sample, which correspond to the sodalite heterophase in the phase.

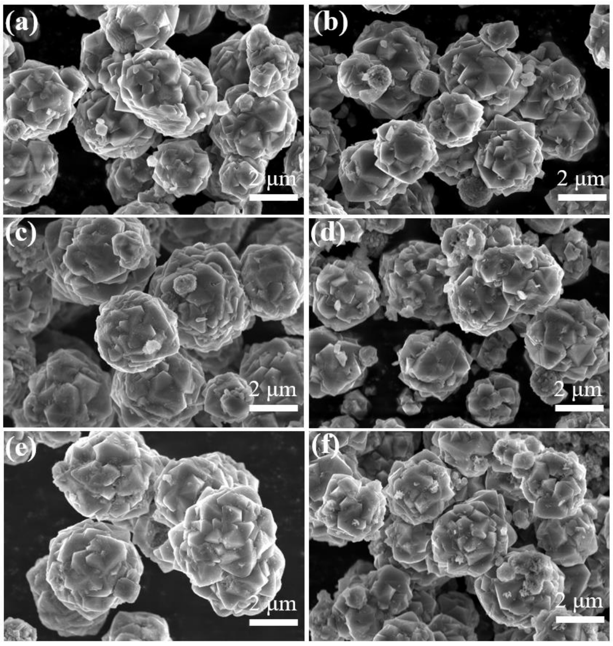

Figure S4. SEM images of KNaLSX samples at different crystallization times

(a) 0.5h (b) 1h (c) 1.5h (d) 2h (e) 2.5h (f) 3h

The **sy**nthetic samples with different crystallization times have typical octahedral structure with an average diameter of 3~4 μm. There are a few tetrahedral small grains in the samples with crystallization time 0.5 and 1h, and NaA heterophase in the corresponding phase. With the increase of crystallization time, the small grains disappear, the crystallinity of the sample increases, the particle size is uniform, and the small grains are attached around the sample for 3h, corresponding to the sodalite heterophase in the phase.

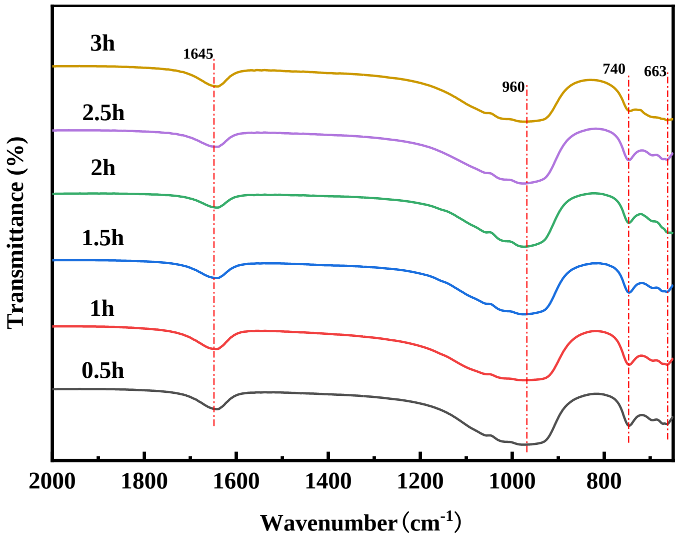

Figure S5. FT-IR patterns of KNaLSX samples at different crystallization times

The absorption peaks at 663, 740 and 960 cm^{-1} correspond to the symmetric and asymmetric stretching vibration of TO4 (T=Si or Al) tetrahedron, and the bending vibration and stretching vibration absorption peaks at 1645 cm^{-1} correspond to the physical adsorption of water and the surface hydroxyl group. The infrared spectra of samples with different crystallization times are consistent. Although there are impurities presented in the KNaLSX samples at crystallization times 0.5, 1 and 3h, the content is very low, which can be seen from Figure 4(a).

Table S2 Properties of LSX zeolites synthesized from industrial waste

Raw material	The proportion of X-type zeolite (%)	Crystallinity (%)	SiO_2/Al_2O_3	Particle size (μm)	S_{BET} (m^2/g)	Reference
Rice husk ash	100	-	2.18	3~6	499	[1]
Fly ash	41	-	2.06	0.7~4	-	[2]
Coal gangue	100	-	2.10	4~5	634	[3]
	-	-	-	-	401	[4]
Potash feldspar	100	100	2.08	5~8	467.8	[5]
	100	-	-	-	-	[6]
	＞90	100	~2.0	2~6	-	[7]
Kaolin	100	93.46	-	4~5	-	[8]
	100	100	-	4~13	-	[9]
Lithium slag	63.7	-	2.2	4~5	632	[10]
	100	88.83	2.01	3~4	714	This work

Reference

[1] Tontisirin S. Synthesis and characterization of co-crystalline zeolite composite of LSX/A. *Micropor. Mesopor. Mat.* **2017**, *239*, 123-129.

[2] Adamczyk Z.; Cempa M.; Białecka B.; Synthesis of Na-LSX type zeolite from Polish fly ash. *Gospod. Surowcami. Min.* **2020**, *36*, 145-166.

[3] Liu, C.H.; Rao, F.; Guo Y.L.; et al. CO_2 capture using low silica X zeolite synthesized from low-grade coal gangue via a two-step activation method. *J. Environ. Chem. Eng.* **2024,** *12*, 112074.

[4] Zang Y.; Wang X.L.; Wang S.Y. Preparation of LSX zeolite from coal gangue in Wuhai area. *China Powder Sci. Technol.* **2019**, *25*, 118613.

[5] Han Z.Y.; Gou M.L.; Chen Z.W.; et al. Synthesis of low silicon aluminum ratio X molecular sieve from potassium feldspar and its ion exchange property. *J. Chin. Ceram. Soc.* **2023**, *51*, 2978-2985.

[6] Bai Y.; Zhao B.; Miao Q.Y.; et al. Activation of the potassic rocks by mixed alkali fusion method and synthesis of low silica X zeolite. *Non-Metallic Mines* **2016**, *39*, 20-23+53.

[7] Miao Q.Y.; Zhao B.; Liu S.J.; et al. Decomposition of the potassic rocks by sub - molten salt method and synthesis of low silica X zeolite. *Asia Pac. J. Chem. Eng.* **2018**, *57*, 8381-8387.

[8] Luo H.J.; Zhou T.; Zhao W.N.; et al. Hydrothermal synthesis of high purity LSX zeolite with coal-measure kaolin. *Multipurpose Util. Miner. Resour.* **2012**, *5*, 32-34+49.

[9] Basaldella E.I.; Tara J.C. Synthesis of LSX zeolite in the NaK system: Influence of the NaK ratio. *Zeolites* **1995**, *15*, 243-246.

[10] Outram, J.G.; Collins F.J.; Millar G.J.; et al. Process optimisation of low silica zeolite synthesis from spodumene leachate residue. *Chem. Eng. Res. Des.* **2023**, *189*, 358-370.
